# CaRE @ Home: Pilot Study of an Online Multidimensional Cancer Rehabilitation and Exercise Program for Cancer Survivors

**DOI:** 10.3390/jcm9103092

**Published:** 2020-09-25

**Authors:** Anne Marie MacDonald, Aleksandra Chafranskaia, Christian J. Lopez, Manjula Maganti, Lori J. Bernstein, Eugene Chang, David Michael Langelier, Maya Obadia, Beth Edwards, Paul Oh, Jacqueline L. Bender, Shabbir MH Alibhai, Jennifer M. Jones

**Affiliations:** 1 Cancer Rehabilitation and Survivorship Program, Princess Margaret Cancer Centre, Toronto, ON M5G 2C1, Canada; annemarie.hospod@uhnresearch.ca (A.M.M.); aleksandra.chafranskaia@uhn.ca (A.C.); christian.lopez@uhnresearch.ca (C.J.L.); lori.bernstein@uhn.ca (L.J.B.); eugene.chang@uhn.ca (E.C.); david.langelier@uhn.ca (D.M.L.); maya.obadia@uhnresearch.ca (M.O.); beth.edwards@uhnresearch.ca (B.E.); jackie.bender@uhnresearch.ca (J.L.B.); 2 IMS Program, Faculty of Medicine, University of Toronto, Toronto, ON M5S 1A8, Canada; 3 Department of Physical Therapy, University of Toronto, Toronto, ON M5G 1V7, Canada; 4 Department of Biostatistics, Princess Margaret Cancer Centre, Toronto, ON M5G 2C1, Canada; Manjula.Maganti@uhn.ca; 5 Department of Psychiatry, Faculty of Medicine, University of Toronto, Toronto, ON M5S 1A8, Canada; 6 Department of Medicine, University of Toronto, Toronto, ON M5S 1A8, Canada; paul.oh@uhn.ca (P.O.); shabbir.alibhai@uhn.ca (S.M.A.); 7 Department of Supportive Care, Princess Margaret Cancer Centre, University Health Network, Toronto, ON M5G 2C1, Canada

**Keywords:** cancer survivorship, cancer rehabilitation, behavior change, digital intervention

## Abstract

Background: Although facility-based cancer rehabilitation and exercise programs exist, patients are often unable to attend due to distance, cost, and other competing obligations. There is a need for scalable remote interventions that can reach and serve a larger population. Methods: We conducted a mixed methods pilot study to assess the feasibility, acceptability and impact of CaRE@Home: an 8-week online multidimensional cancer rehabilitation and exercise program. Feasibility and acceptability data were captured by attendance and adherence metrics and through qualitative interviews. Preliminary estimates of the effects of CaRE@Home on patient-reported and physically measured outcomes were calculated. Results: A total of *n* = 35 participated in the study. Recruitment (64%), retention (83%), and adherence (80%) rates, along with qualitative findings, support the feasibility of the CaRE@Home intervention. Acceptability was also high, and participants provided useful feedback for program improvements. Disability (WHODAS 2.0) scores significantly decreased from baseline (T1) to immediately post-intervention (T2) and three months post-intervention (T3) (*p* = 0.03 and *p* = 0.008). Physical activity (GSLTPAQ) levels significantly increased for both Total LSI (*p* = 0.007 and *p* = 0.0002) and moderate to strenuous LSI (*p* = 0.003 and *p* = 0.002) from baseline to T2 and T3. Work productivity (iPCQ) increased from T1 to T3 (*p* = 0.026). There was a significant increase in six minute walk distance from baseline to T2 and T3 (*p* < 0.001 and *p* = 0.010) and in grip strength from baseline to T2 and T3 (*p* = 0.003 and *p* < 0.001). Conclusions: Results indicate that the CaRE@Home program is a feasible and acceptable cancer rehabilitation program that may help cancer survivors regain functional ability and decrease disability. In order to confirm these findings, a controlled trial is required.

## 1. Introduction

Due to advances in early detection and biomedical treatment, over two-thirds of people who are diagnosed with invasive cancer will become long-term survivors [1,2]. Cancer survivors have unique post-treatment needs and face multiple physical, functional, and psychosocial challenges as they transition back to their lives [3,4,5,6,7,8]. Cancer rehabilitation is an essential component of survivorship care and has become increasingly relevant as the number of cancer survivors grows, coupled with the high documented rates of physical impairment and disability [9,10,11,12]. Comprehensive cancer rehabilitation focuses on prevention and treatment of immediate, persistent, or late effects of cancer and treatment, and the maintenance of health to optimize functional status and quality of life (QoL) [11,13,14]. Embedding cancer rehabilitation as a standard part of cancer care has the potential to improve patient outcomes and reduce the burden on the healthcare system [9]. For patients with complex rehabilitation needs, a personalized, coordinated multidisciplinary approach is required to decrease disability and achieve improvements in physical functioning and overall QoL.

To date, the evidence on cancer rehabilitation is derived largely from trials utilizing face-to-face delivery in a clinical setting [9,14]. However, notwithstanding the advantages of facility-based supervised interventions [15], cancer survivors face significant barriers (e.g., time, work, remote home locations, cost, poor health) that can prevent access to cancer rehabilitation services delivered in medical facilities. Many cancer survivors prefer distanced-based interventions because of removed costs and travel to a medical facility, and the ability to accommodate competing commitments [16]. The rapid growth of eHealth technology has led to the development of new applications that can enable health care providers to monitor, treat, educate and support patients in their own homes. Telerehabilitation interventions that harness current and emerging technologies have been suggested as one mechanism that can reduce some barriers to accessing and providing rehabilitation [17,18]. This approach can allow for cost effective disease management and health promotion and is well established in other chronic disease populations such as heart disease and diabetes [19,20,21,22,23]. Specific to cancer survivors, eHealth technology presents opportunities to increase access to cancer rehabilitation in a virtual setting [24] and has shown promise in increasing physical activity [25,26] and reducing specific psychosocial and physical symptoms [27,28,29,30,31,32]. In one recent study, Cheville and colleagues [33] assessed the effect of a telerehabilitation intervention (including remote monitoring of symptoms and an individualized physical conditioning program) on function in patients with advanced cancer and found that the intervention improved function and pain, and decreased hospital length of stay and the requirement for post-acute care. However, impairment-driven multidimensional cancer telerehabilitation interventions have yet to be developed and evaluated for their feasibility, acceptability, and impact. Research on the development and impact of cancer telerehabilitation is urgently needed and should include feedback from patients to improve the quality of the rehabilitation itself [23].

We recently developed and implemented a virtual 8-week impairment-driven multidimensional Cancer Rehabilitation and Exercise program (CaRE@Home) with the aim to restore and optimize function and well being in those with identified cancer-related impairments. The aim of this mixed-methods pilot study was to assess the feasibility, acceptability, and preliminary impact of the CaRE@Home program.

## 2. Methods

We conducted a single-arm explanatory sequential mixed methods pre-post-assessment of the CaRE@Home program in cancer survivors with moderate-high disability at Princess Margaret Cancer Centre in Toronto Canada. The research aims were to assess: (1) feasibility; (2) acceptability; and (3) to gain a preliminary estimate of the effects on disability (primary outcome), physical symptoms, social functioning, distress, physical activity, work function, and physiological factors. Outcomes were assessed at baseline (T1), immediately post-8 week intervention (T2) and 3 months post-intervention (T3). A sub-sample of participants were invited to take part in a post-intervention (T3) qualitative interview to explore participant experience of the program and gather feedback. The study was approved by the University Health Network Research Ethics Board, with participants providing written informed consent.

### 2.1. Intervention

The Cancer Rehabilitation and Survivorship (CRS) Program at Princess Margaret Cancer Centre provides impairment-driven cancer rehabilitation to patients during and after acute cancer treatment with the goal to minimize disability and maximize functional independence. As part of its programming, CRS offers an innovative and effective in-person 8-week structured group multidimensional program, which is delivered at the Centre for Health Wellness and Cancer Survivorship (ELLICSR) at the Toronto General Hospital. This in-person group program, Cancer Rehabilitation, and Exercise (CaRE@ELLICSR), consists of weekly group exercise classes and self-management skills education delivered by CRS rehabilitation experts. Data collected on the in-person CaRE@ELLICSR intervention have demonstrated significant improvements in disability, social functioning, distress, and physical activity, and extremely high ratings of participant satisfaction [34]. However, ~40% of referred patients decline participation due to travel time, transportation costs, or competing obligations. In response, in collaboration with the Princess Margaret Educational Design & Knowledge Translation program and using a series of iterative steps proposed by the NCI Research-Tested Intervention Programs (NCI 2019), we adapted the CaRE@ELLICSR program so that it could be delivered through a virtual platform (CaRE@Home).

CaRE@Home is an 8-week program comprised of: (1) individualized progressive exercise prescription supported with a mobile application (Physitrack^®^) and wearable technology (Fitbit™) to track activity; (2) weekly e-modules providing interactive education to promote self-management skills; and (3) weekly brief telephone health coaching provided by health professionals (e.g., kinesiologists) trained in motivational interviewing. Informed by behavior change theory, the program components aim to provide patients with the knowledge and tools needed to reach and maintain their wellness and exercise goals (see Table 1 for list of embedded behavior change techniques and tools [35]). Multiple theoretical models are integrated within our intervention (i.e., motivational interviewing, cognitive behavioral therapy, theory of planned behavior), with the focus on addressing and resolving motivational ambivalence and identification and modification of the cognitive distortions that prevent adoption of appropriate health behaviors and addresses relapse and long term maintenance of behavior change [36,37,38,39,40,41]. The program is free to participants, other than out of pocket travel costs (i.e., public transport, parking) for in-hospital assessments.

1. Exercise prescription, Physitrack^®^ and Fitbit™: Prior to beginning the program, participants underwent an in-person fitness assessment conducted by a registered kinesiologist (RKin). Based on this assessment, an individualized exercise prescription was developed and progressed towards the ACSM guidelines of 150 min per week of moderate intensity aerobic exercise, 2 to 3 days of resistance training, and routine large muscle group flexibility training [42,43]. (Updated guidelines were published in 2019.) The individualized exercise prescription was then entered by the RKin into Physitrack^®^, an online platform that allows customizable exercise prescriptions, tracking of exercise completion, and video tutorials to support the prescribed exercises. In addition, participants were provided with a Fitbit™ device, which serves as a self-monitoring tool and provides further assistance to participants in adhering to recommended physical activity routines and tracking physical activity. Data from the device was also used as a weekly monitoring tool by the health care professionals. The participants were provided with an orientation to the Physitrack^®^ application and Fitbit™ device during their initial in-person fitness assessment.
2. E-Learning Modules: Participants were registered into an online learning platform and completed 8 comprehensive self-management e-modules. Modules were locked, meaning they had to complete the prior week before unlocking the next, and took approximately 20–45 min to complete. These modules included: Week 1: Getting Started—Set Your Goals; Week 2: Eat and Cook for Wellness; Week 3: Reduce Fatigue; Week 4: Manage your Emotions; Week 5: Be Mindful; Week 6: Improving Cancer-related Brain Fog and Boosting Your Brain Health; Week 7: Find Ways to Connect and Week 8: Plan for the Future. The topics chosen were based on patient-reported needs and commonly presenting symptoms. The content was developed by a multidisciplinary cancer rehabilitation team, including occupational therapy, physiotherapy, dietetics, neurocognitive psychology, social work, kinesiology, physiatry, and also included behavioral scientists. The modules included health literate design features such as linear navigation, intuitive buttons, and plain language and underwent two rounds of usability testing prior to the launch of the program. Attention was placed on behavior change techniques, ensuring that the interactive elements strengthened any behavior change goals.
3. Telephone Health coaching: Participants received one weekly health coaching call with the same health coach in weeks 2–7 (6 total) plus one maintenance call at 1 month and one last call at 2 months post-intervention. Calls were scheduled for 20 min. Coaches were either a RKin or social worker trained in behavioral change and utilizing motivational interviewing skills. They guided participants to reflect upon their last week, plan goals for the week ahead, and identify barriers and solutions in achieving their goals and incorporated assessment and enhancement of motivation, promotion of self-efficacy, and collaborative problem solving. The objective was to enhance sustained behavior change by integrating several active ingredients outlined in the cancer-survivor behavior change literature [44,45] and motivational interviewing (MI) [36]. MI is a collaborative counseling method that elicits and strengthens motivation for change by addressing and resolving ambivalence [46] and has been shown to be effective in increasing physical activity in cancer survivors and those with other chronic conditions [47,48,49,50,51,52]. Health coaches were trained in motivational interviewing and supervised by a certified Motivational Interviewing Network Trainer who was part of the team (MO).

### 2.2. Participants and Procedure

Potential participants were recruited from the CRS program at Princess Margaret Cancer Centre and identified during their initial comprehensive rehabilitation assessment with a physiatrist and an occupational or physical therapist. Participants who met inclusion criteria were given an information pamphlet describing the program, as well as a patient agreement form that detailed both patient and health care provider responsibilities and expectations. Eligibility criteria included: age ≥ 18 years; received clearance by the CRS physiatrist to participate in exercise; expressed comfort using technology; ability to attend the in-person initial, 8 week, and 3 month follow-up assessments; fluency and/or comfort with the English language. Participants were excluded if they had acute medical needs that required a referral to another specialized service (i.e., Cancer Pain Program); had a planned surgery during the timeline of the study; presented with only mild impairments that could be managed by community partners; or had incurable cancer.

Eligible and interested patients were contacted by the CaRE intake coordinator to schedule their initial fitness assessment. At the initial fitness assessment, participants completed the questionnaire package using a tablet and then met with a team RKin for the fitness assessment. During the appointment, they were on-boarded onto the eLearning platform and Physitrack^®^ application, provided with the Fitbit^TM^, and weekly health coaching calls were scheduled. Consent was obtained at the baseline assessment. Participants also completed the questionnaire package and fitness assessment at the end of the 8-week program (T2) and 3 months later (T3). Qualitative interviews were conducted after T3 with a sub-sample of participants. Participants who had completed the 8-week program and had consented to be contacted for interview purposes were contacted by phone or email. Interviews were scheduled after participants had completed their 3-month follow-up assessment.

### 2.3. Study Outcomes

#### 2.3.1. Demographic and Clinical Data

Basic demographic and clinical information were obtained by chart review at baseline and from the initial assessment questionnaire. Data included age, sex, marital status, employment status, time since diagnosis, and primary cancer location.

#### 2.3.2. Feasibility and Acceptability

Feasibility of the intervention and methods were assessed by tracking the following: (1) Recruitment rates defined by the number of participants who completed an in-person baseline assessment compared to the number who were referred; (2) Retention rates took into account the patients who participated in the 8 week intervention and completed the follow-up assessments at 8 weeks and 3 months. (3) Adherence was examined by health coaching call attendance, Fitbit™ and Physitrack^®^ usage, and e-module completion. To further assess feasibility and to evaluate acceptability and steer future program refinement, we conducted in-depth semi-structured qualitative telephone interviews with a sub-sample of participants following the T3 assessment. All interviews were conducted by the same person (BE), a trained qualitative methodologist. Interviews lasted between 20–45 min in length.

#### 2.3.3. Exploratory Clinical Outcomes

Questionnaire and fitness assessment data were collected to measure the program’s effect on disability (primary outcome), symptom severity, physical activity, work function, and physiological factors (secondary outcomes). Data were collected at baseline (T1), immediately post-intervention (8 weeks, T2) and 3 months post-intervention (T3).

1. Disability was measured using the 12-item World Health Organization’s Disability Assessment Schedule 2.0 (WHO-DAS 2.0) [53,54]. Respondents rate their difficulty in engaging in particular activities on a scale from “none” (no difficulty) to “extreme or cannot do” on six domains of functioning. Scores range from 12 to 60, where higher scores indicate higher disability or loss of function. A WHODAS score of 0–4 is defined as none to mild disability and 5–48 is defined as moderate/high.
2. Symptom Severity was measured with the widely used Edmonton Symptom Assessment Schedule revised (ESAS-r) [55,56]. The ESAS-r includes a simple 0–10 severity rating scale (0 being ‘symptom is absent’ and 10 being ‘worst possible severity’) for nine symptoms common in cancer patients: pain, tiredness, nausea, depression, anxiety, drowsiness, appetite, wellbeing, and shortness of breath.
3. Physical Activity was measured using the Godin-Shephard Leisure-Time Physical Activity Questionnaire (GSLTPAQ) [57]. The GSLTPAQ is a 3-item questionnaire that includes three questions on the number of times respondents engage in mild, moderate and strenuous leisure time physical activity (LTPA) bouts of at least 15 min duration in a typical week. Participants also report on the average minutes of each session for each intensity level. Examples of LTPA for each intensity category are provided. A Leisure Score Index (LSI) is calculated by taking the number of bouts at each intensity and multiplying by 3, 5, and 9 metabolic equivalents (METs) and then summing the score. In addition, moderate, and strenuous LTPA can be summed and used to categorize respondents as active and insufficiently active based on physical activity guidelines for cancer survivors.
4. Work Function was assessed using two items from the iMTA Productivity Cost Questionnaire (iPCQ). Absenteeism was measured in numerical hours by asking participants: “During the past seven days, how many hours did you miss from work because of problems associated with cancer diagnosis and treatment?” Productivity was assessed by asking participants: “During the past seven days, how much did your cancer diagnosis and treatment affect your productivity while you were working?” with answers measured on a 0 to 10 scale, with 0 = problem had no effect on my work and 10 = problem completely prevented me from working [58].
5. Physiological measures were collected by the RKin during the in-person assessments and included aerobic capacity and endurance (6-min walk test) [59,60,61]; strength (grip strength) [62], and; cardiometabolic health (body mass index (BMI), resting heart rate and blood pressure).

### 2.4. Data Analysis

The target sample size for this pilot study (*n* = 30–40) was based on our primary aim to assess feasibility of the methods and acceptability of the intervention and is consistent with sample size recommendations for behavioral therapies research [63].

#### 2.4.1. Feasibility and Acceptability

The proportion of patients who were referred to CaRE@Home and who enrolled into the program and provided consent (recruitment rate) and the attrition rates at each time point were calculated. Feasibility was assessed by calculating participant adherence to the 6 health coaching calls that took place during the 8-week intervention (weeks 2–7 inclusive), to the 8 weekly e-modules, and to Fitbit™ and Physitrack^®^ usage.

Qualitative interviews were completed to assess feasibility and acceptability. A semi-structured interview guide was developed based on previous CaRE evaluation scripts and revised to address feasibility and acceptability issues specific to CaRE@Home (i.e., health coaching, technology, home-based exercises, online modules). All interviews were digitally recorded and transcribed verbatim. Transcripts were first read multiple times in order to understand the participants’ perspectives and experiences. Our analysis began deductively, with a select number of categories pre-selected to align with the main program components—for example exercise prescription, educational modules, and health coaching calls. Acceptability categories were also pre-selected, namely program strengths, weaknesses, and areas of improvement. The transcripts were first annotated for these categories. Once this initial coding was complete, the interviews were then read once again, and codes were derived inductively, meaning that they were derived from the data and not preselected. Themes were generated by a close examination of codes and categories, and the relationships between them, and discussions with the research team. The main categories are related to the study objectives, were discussed consistently within an individual interview, as well as those that were discussed repeatedly between participant interviews. We sought to answer the questions of why the participants liked or disliked certain program components, how they engaged with the program, and in what ways the program impacted their lives [64]. Illustrative quotes were chosen based on their connection to these categories and themes, and whether they provided valuable insight for future program iterations. Recruitment for interviews was stopped once data saturation was reached and no new categories, codes, or themes were emerging.

#### 2.4.2. Exploratory Clinical Outcomes

Demographic and clinical data were reported using descriptive statistics. Continuous data were reported as means with standard deviations and categorical data was reported as percentages. Statistical analyses were performed on data from the participants who completed baseline measures. For all exploratory clinical outcomes that are continuous in nature, estimated means at each time point were calculated and compared using linear mixed effects models. The proportion of patients with moderate to high disability (WHODAS 2.0) was examined across time points using GEE (Generalized Estimating Equations) procedures and the corresponding *p*-value was reported. All statistical analyses were conducted using SAS Version 9.3 or R version 3.5.1. Statistical significance is considered as *p* < 0.05.

## 3. Results

A total of *n* = 35 participants enrolled in the study. Participant demographic data are presented in Table 2.

### 3.1. Feasibility

**Figure 1:**
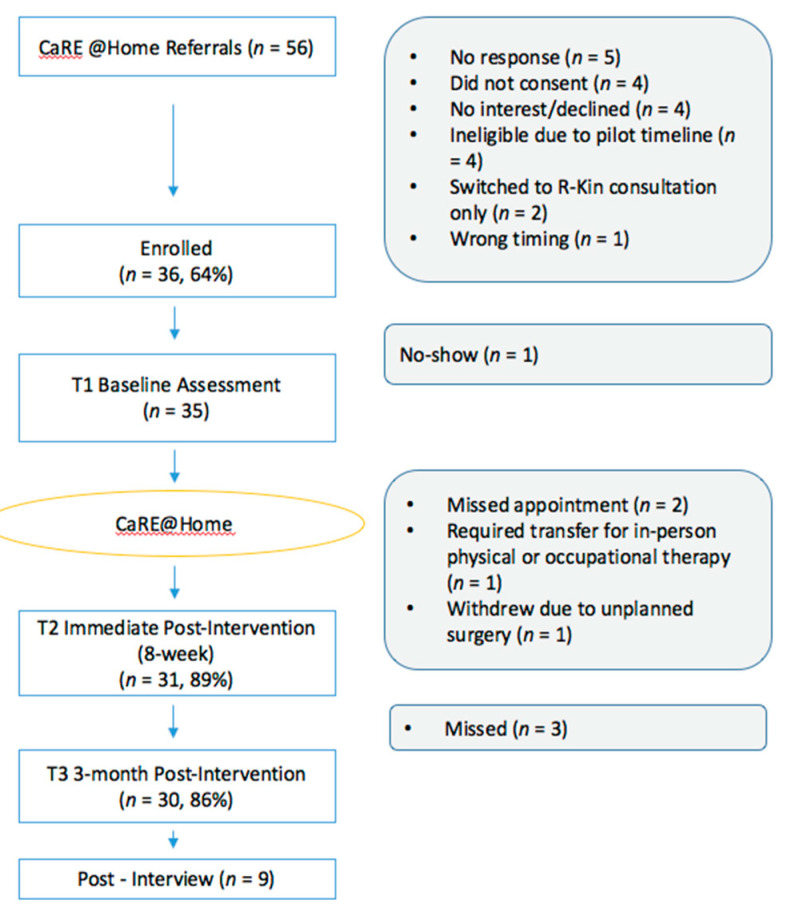
Program flow.

The study flow diagram is presented in Figure 1. Recruitment took place between May 2019 and October 2019 during which time 56 patients were identified as eligible for CaRE@Home and referred for booking and 51 were successfully contacted by the CaRE coordinator. Of these, 5 patients declined participation since they were no longer interested in joining, 6 were deemed ineligible due to inability to attend the assessment appointments, and 4 did not provide consent to be part of the study. A total of 35/36 scheduled baseline assessments (T1) were completed, with one patient not showing up for the scheduled assessment. Thirty-one participants completed the 8 week T2 assessment, and 30 patients completed the T3 assessment at 3-month post-intervention. The recruitment rate was 64% (36/56) and retention rate was 83% (30/36).

Feasibility was also measured by measuring adherence to health coaching calls, Fitbit^TM^ and Physitrack^®^ usage, and e-module completion. Participants were pre-scheduled for 6 health coaching calls during weeks 2–7 of the 8-week intervention (inclusive). Call adherence ranged from 1–6 calls with an average of 5 calls. A total of 17/35 (49%) participants completed all six calls, with 28/35 (80%) participants completing at least 5 calls. Following the 8-week program, participants had two maintenance calls at 1 month and 2-months post-intervention. Twenty-five calls (71%) were completed at the 1-month mark and twenty-nine calls (83%) were completed at the 2-month mark. Fitbit^TM^ usage was measured as the percentage of days that the device was worn throughout the 8-week intervention (total intervention length ranged from 53 to 65 days due to scheduling variability for T2 assessment). In total, 30/35 (86%) participants received and wore the device. Some participants did not want the Fitbit^TM^ since they already had their own device (*n* = 3). Login details were missing or changed for 2 participants and as such Fitbit^TM^ data could not be downloaded for them. Of those who received the device and for whom login information was available, the device was worn for 23%–100% of the intervention days, with a mean of 87% of the intervention days. Thirty-one participants (89%) logged into the Physitrack^®^ app at least once throughout the intervention. Adherence to the e-modules was measured by the completion rate of the 8 weekly e-modules. Twenty-seven of the 35 participants logged into the online learning portal (77%) at least once, while 7 participants never logged on at all. Module adherence ranged from 0–8 modules, with a mean of 4 modules completed, and a mode of 0 or 6 modules completed by 5 participants. Participants were most likely to complete the first module (22/27; 81.5%), with a weekly drop-off occurring thereafter, and only 4/27 (14.8%) completed the final eighth module.

Data from the qualitative interviews (*n* = 9) were analyzed for feasibility issues. A sample of 10 participants who completed their 3-month follow up assessment was selected for qualitative interviews. Nine interviews were scheduled and completed during the pilot study. The findings suggest that some aspects of the program structure were well received and reasonable for our patient population. Participants also identified areas of improvement (see Table 3). Most participants found the program to be well organized and structured to suit their needs. They appreciated the remote program nature but also valued the weekly check-ins with the health care professional. They found the program to be flexible and convenient and liked being able to complete the program from the comfort of their home. Others spoke of the benefit of not having to be a part of a group setting, where they often felt uncomfortable. Some participants shared that the program length and follow-up frequency were appropriate, whereas others shared that the program could have been longer. Lastly, a few participants talked about how they would have preferred more time to complete the e-modules or the ability to have all modules be unlocked (vs. a prescribed weekly order).

### 3.2. Acceptability

Data from the qualitative interviews, were analyzed to assess acceptability. Three key categories emerged. These included overall satisfaction with program and program elements, benefits of the program, and areas for improvement. A summary of these categories is presented below. A detailed description of each of these categories, along with illustrative quotes, is summarized in Table 4.

#### 3.2.1. Overall Satisfaction with CaRE@Home Program

The participants who were interviewed expressed that they felt grateful to have taken part in the program and appreciated the support it provided. Participants saw a need for such rehabilitation programs since acute oncology care was often focused on medical needs whereas this program was more holistic in nature. Feedback from the interviews highlighted that the health coaching calls were a valuable program component that encouraged accountability and provided an appreciated human touch element and support during a time when they often felt alone. The interview findings support the use of technology as an add-on monitoring and motivational tool. The Fitbit^TM^ device was viewed as a valuable self-monitoring tool that motivated participants to move and achieve their target steps and target heart rates. The Physitrack^®^ software enabled patients to have an accessible and visual reminder of their exercise prescription.

#### 3.2.2. Benefits of the Program

Participants spoke about the benefits of the CaRE@Home program including their ability to self-manage their emotions and exercise. Participants spoke about the positive impact of the educational content provided by the e-modules and appreciated the focus on self-care. Many talked about gaining a new appreciation for exercise. Participants also indicated they were able to gain insight and skills on mindfulness and better manage their emotions. As a result of the program, some participants felt that they were better able to accept their diagnosis and the emotions surrounding it.

#### 3.2.3. Areas for Program Improvement

Some participants provided suggestions for improvements to the program. For example, participants had mixed feelings towards the weekly e-modules. While they enjoyed the educational curriculum and found the material to be timely and appropriate, some felt they were too long. In some cases, even though the health coaches would remind participants to complete their modules, participants said they didn’t have enough time to do so. Others expressed that they would have liked to have more time to digest the weekly learnings before moving on and would prefer to be able to pace themselves through the e-modules. A few participants appeared to be more focused on their exercise component of the intervention and less so on the e-module offering. Participants valued the in-person assessments, but some had difficulty travelling to downtown Toronto to attend these visits and suggested that there be a train-the-trainer model with more remote kinesiologists available to perform the assessments in their community. In addition, participants enjoyed connecting with the kinesiologist, and suggested that some of the phone appointments be done over video conferencing to allow for a more personal touch and also demonstration of the exercises. One participant suggested that there could be some specific programming or content created for the main caregiver at home who is supporting the patient.

### 3.3. Exploratory Clinical Outcomes

Preliminary estimates of the treatment effects for participants on disability (primary), symptom severity, physical activity and work status were evaluated (Table 5). WHODAS 2.0 disability scores significantly decreased from baseline to T2 and T3 (*p* = 0.03 and *p* = 0.008). Further, the proportion of participants reporting moderate/high levels of disability decreased from 75.8% at baseline to 50% at T3 (*p* = 0.039). There were no differences observed from baseline to T2 or T3 for any of the symptom severity (ESAS) measures. The GODIN-Shephard Leisure-Time Physical Activity Questionnaire revealed a significant increase in Total LSI (*p* = 0.007 and *p* = 0.0002) and moderate to strenuous LSI (*p* = 0.003 and *p* = 0.002) from T1 to T2 and T3. Finally, there was an improvement in productivity with a significant decrease in WS6 productivity scores (During the past seven days, how much did your cancer diagnosis and treatment affect your productivity while you were working?) from T1 to T3 (*p* = 0.026). There seems to be a decreasing pattern in WS3 absenteeism scores (During the past seven days, how many hours did you miss from work because of problems associated with cancer diagnosis and treatment) from baseline to T2 and T3, but it did not reach statistical significance.

In addition, preliminary estimates of the treatment effects on physiological outcomes were evaluated (Table 6) There was a significant increase in six-minute walk distance from baseline to T2 and T3 (*p* < 0.001 and *p* = 0.010) and in grip strength from baseline to T2 and T3 (*p* = 0.003 and *p* < 0.001). No differences were observed from baseline to T2 or T3 for heart rate, diastolic or systolic blood pressure, or BMI. 

## 4. Discussion

This pilot study generated valuable findings regarding the CaRE@Home intervention and suggests that its delivery and method of evaluation are feasible, and it is acceptable to participants. In addition, we found statistically significant improvement in disability scores at 3 months and also improvements in physical activity, work productivity, grip strength, and 6-min walk distance. The CaRE@Home intervention was innovative in that it was impairment-driven and included: (1) individualized progressive exercise prescription supported with a mobile application and wearable technology; (2) weekly e-modules providing interactive education to promote self-management skills; (3) weekly brief telephone health coaching with a member of the rehabilitation team; and (4) was informed by behavior change theory.

Overall, the CaRE@Home program was feasible with high rates of retention in the program. The 83% retention rate was extremely high given this type of intervention [65,66]. Participants enjoyed the flexibility that a remote program provided and appreciated the ability to only come into the hospital for select appointments and follow-ups. Participants particularly liked working directly with their assigned health coach and receiving custom exercise prescriptions tailored to their functional abilities. They also found the weekly health coaching calls very helpful, noting that the support and accountability motivated them to engage with the program and complete their exercise prescriptions. This finding is supported by other research, which has shown that having external accountability is an important facilitator to behavior change [67]. Telerehabilitation has been shown to strengthen the patient-health-care provider connection by enhancing the provider’s knowledge of the patient and their contextual factors, providing ongoing information exchange and facilitating education, and establishing shared goal setting and action planning [68].

The use of self-monitoring technology was a key factor that supported the participants’ adherence to the program. Use of both the Fitbit™ and Physitrack^®^ application were extremely high. Participants appreciated the ability to have their custom exercise prescriptions updated on Physitrack^®^ as their strength and functional abilities changed. Patients also found the Fitbit™ device to be motivating and to provide nudges to reach their daily goals. The use of self-monitoring devices such as the Fitbit™ device has been shown in multiple studies to motivate participants to take on healthy active behaviors, when paired with additional behavior change interventions [69,70,71,72]. Fitbits™ have been found to be especially beneficial when used in conjunction with other program elements [71]. Results from our pilot study also indicate that exercise-based oncology programs should consider including technological tools so as to provide effective and accessible options to a greater patient population. The use of technology may also make it easier for intervention-deliverers (exercise and rehab professionals) to prescribe exercise and monitor patients’ needs.

A Web-based educational curriculum is also an important behavior change element as it shapes knowledge through a highly accessible format [35]. Most of the pilot participants (22/27; 81.5%) engaged with the online content and completed at least the first module. However, participants completed an average of only 50% of the modules. We observed a significant drop-off occurring week to week with only 4/27 (14.8%) completing the last module in week 8 of the intervention. From the qualitative interviews we learned that many participants appreciated the value of the modules but did not prioritize their completion, and many felt they were too long and that they did not have sufficient time to invest in completing them. The modules were also prescribed weekly and locked, requiring the completion of the current module to unlock the next. Participants who fell behind did not have the option to skip ahead and may have found this prescriptive approach too difficult to manage or not meeting their needs or interest. Future iterations of the program will effectively “unlock” modules to allow for more flexibility and will include automated weekly email reminders to complete the e-modules (including the time required and the goals of the module).

Digital and remote program development is often iterative in its implementation [73]. Pilot studies, such as this one, are invaluable in generating acceptability data for future, larger scale trials. Our pilot findings support the acceptability of a remote cancer rehabilitation and exercise program, but also brought to light important areas for program improvements. Participants felt that this program should also be offered during treatment, as much of the content would have been beneficial. As mentioned above, some participants also suggested that all modules be readily available during the 8-week intervention, rather than have a prescribed weekly module. Suggestions were also made in regards to content; some participants suggested adding in content and support for the patient’s primary caregiver, something which has been known to be beneficial in other studies [74,75]. Others found the modules to be too long. Participants valued the in-person assessments and the health coaching calls so much so that they suggested having more video health coaching in order to improve the human touch element of the intervention. Lastly, some participants suggested a train-the-trainer model with remote kinesiologists who could perform the in-person assessments in the community, rather than have patients come to downtown Toronto.

Finally, while distance-based eHealth interventions have the potential to reduce disparities among cancer survivors and improve access [76,77] and the large majority of adults have access to the internet and smart phones [78,79], it is important to recognize that there are a number of cancer survivors who face barriers to accessing eHealth interventions. This may include those with cognitive difficulties, limited technological literacy, physical disabilities, and for whom English is not their first language. Given this, it is important to identify cancer survivors with particular needs when introducing health technology and to develop strategies to prevent leaving behind this substantial group of cancer survivors [80,81]. In the current study, only 64% of those who had been identified as potentially eligible for this program actually enrolled. It is important for clinicians to assess an individuals’ receptiveness to use technology within a rehabilitation context in order to tailor the interventions offered [81].

### Study Limitations

Results from this pilot study need to be interpreted while acknowledging its limitations. To begin, while the sample size was appropriate for a pilot study, the impact of the program on clinical outcomes needs to be considered preliminarily. Further, as this was a one-arm study with no control condition, there may be other explanations for improved disability (e.g., elapsed time since treatment) that must be considered. Encouragingly, the qualitative results supported the quantitative findings. Secondly, follow up assessments were limited to 3 months post-intervention, which does not allow for the examination of the long-term impact of the intervention or maintenance of health behaviors. Intervention components may show greater impact after a longer duration and thus reporting of outcome measures at 6 and 12 months would make for more comprehensive results. In addition, while results from the qualitative study demonstrate a high degree of acceptability, it is important to note that the interviews were conducted with participants who completed the study; completing a mean of 4 modules (range of 0–8) and wore the Fitbit^TM^ for a mean of 84% of the intervention days. The perspectives of these individuals may not reflect the perspectives of those who did not complete the intervention, or of those who were less engaged with the e-modules and the Fitbit^TM^ device. It is also worth noting that we were unable to track specific usage of the Physitrack app (other than whether or not participants ever logged in) and so adherence to this aspect of the intervention is limited. Finally, safety incidents were not actively tracked in this pilot study, though none were noted, and should be considered in future trials.

## 5. Conclusions

This study provides important information on the feasibility, acceptability, and impact of a virtual multicomponent cancer rehabilitation intervention for cancer survivors, thus addressing an important gap in the literature. High retention and positive outcomes at 3 months are encouraging indications of the potential success of the CaRE@Home intervention and these results support moving to a Phase III controlled trial.

## Figures and Tables

**Table 1:** Behavior Change elements and tools for CaRE @ Home.

Behavior Change Elements		
Behavior Change Element (BCT Taxonomy)	Care @ Home Component	Notes/Details
Goals and Planning	Goal discussion and agreement during initial assessment Goal setting (Week 1) module completion Weekly action plan completion	Behavior change techniques that are also included here: Problem Solving, Goal setting, Action Planning, Review behavior goal(s), Discrepancy between current behavior and goal, Review outcome goal(s), Behavioral contract, Commitment
Feedback and Monitoring	Weekly action plans Physitrack^®^ self- monitoring Fitbit^TM^ self-monitoring Follow-up feedback and nudges E-module quizzes and exercises	Patients fill out weekly action plans that are reviewed during their follow-up calls. Patients are given feedback and support during their weekly follow-up calls and visits. Behavior change techniques that are also included here: Monitoring of behavior by others without the feedback, Feedback on behavior, Self-monitoring of behavior, Feedback on outcome(s) of behavior
Social Support	Support provided by the program staff and health coach during the various program touch points (assessments, follow-ups)	Behavior change techniques that are also included here: Social support (unspecified), Social support (practical), Social support (emotional).
Shaping Knowledge	E-module Content Education during initial assessment, Follow-Up calls and visits	Behavior change techniques that are also included here: Instruction on how to perform the behavior, Information about antecedents, Re-attribution
Natural Consequences	E-module content and follow-up calls provide information about the consequences of performing the behavior (exercise, self-management behaviors, etc.)	Behavior change techniques that are also included here: Information about health consequences, Monitoring of emotional consequences, Information about emotional consequences
Comparison of Behavior	Physitrack^®^ provides video tutorials of exercises that patients can perform at home	Behavior change techniques that are also included here: Demonstration of the behavior
Repetition and Substitution	Weekly exercise prescriptions with repetitive components Goals are increasingly difficult but achievable	Behavior change techniques that are also included here: Behavioral substitution, Habit formation, Habit reversal, Generalization of a target behavior, Graded Tasks
Reward and Threat	Initial discussion of motivation and self-incentive/self-reward at onset of program Verbal reward or non-verbal (email) reward when certain tasks are completed, or goals achieved	Behavior change techniques that are also included here: Social Reward, Self-incentive, Self-reward
Regulation	Self-management and stress management skills shared throughout the program	Behavior change techniques that are also included here: Reduce negative emotions, Conserving mental resources
Antecedents	Advice is provided on how to change the physical and the social environment in order to facilitate the desired behavior	Behavior change techniques that are also included here: Restructuring the physical environment, Restructuring the social environment, Avoidance/reducing exposure to cues for the behavior
Identity	E-module curriculum Teaching from Kinesiologist/Health coach	Behavior change techniques that are also included here: Identification of self as role model, Framing/reframing, Valued self-identity
Self-belief	Health coach provides reassurance, support, and examples of how to increase confidence in reaching a goal	Behavior change techniques that are also included here: Verbal persuasion about capability, Focus on past success, Self-talk

**Table 2:** Participant Characteristics.

**Category**	*n* (%)
**Sex***n* (%)	
Female	22 (62.9)
Male	13 (37.1)
**Age** (years)	
Mean (SD)	55 (±15.9)
Range	19–76
**Marital Status***n* (%)	
Married/Common Law	25 (71.4)
Other	10 (28.6)
**Education***n* (%)	
High school	7 (20.0)
University/college	25 (71.4)
Missing/Prefer not to answer	3 (8.6)
**Household Income***n* (%)	
<$40,000	2 (5.7)
40,000–75,000	7 (20.0)
>$75,000	14 (40.0)
Missing/prefer not to answer	12 (34.3)
**Employment Status***n* (%)	
Employed (full/part time)	16 (45.7)
Not-employed/on disability/retired	19 (54.3)
**Cancer Site***n* (%)	
Breast	12 (34.3)
Gastrointestinal	5 (14.3)
Lymphoma and Myeloma	5 (14.3)
Gynecological	3 (8.6)
Endocrine	3 (8.6)
Head and Neck	2 (5.7)
CNS	2 (5.7)
Genitourinary	1 (2.9)
Leukemia	1 (2.9)
Lung	1 (2.9)
Time Since Diagnosis (months)	24
mean (SD), range	(±25.4), 5–123
Reason for Referral *n (%)	
Cancer-related Fatigue	20 (57.1)
Deconditioning and Exercise contraindications	19 (54.3)
Return to work limitations	10 (28.6)
Neurocognitive	5 (14.3)
Lymphedema	8 (22.9)
Musculoskeletal	5 (14.3)
Neurological	5 (14.3)
Balance	3 (8.6)
Activities of Daily Living	2 (5.7)

* may be multiple reasons.

**Table 3:** Qualitative Table for Feasibility.

Category	Analytic Note	Example Quote
Program structure and delivery	Participants were satisfied with the program and its structure and glad they took part and found the team very supportive. The program length and frequency were reasonable for most participants, with only a few suggesting that they would have liked a longer program or more frequent check-ins as the program progressed. Participants enjoyed being able to take part in the program from home rather than having to travel to the hospital.	“You know when you have problems that you’re trying to resolve but you can’t by yourself it’s extremely important to have support, and I’m not the kind of person to go and ask for help so the fact that I was offered that in such a friendly and considerate manner was very important emotionally for me.” (Female breast cancer survivor, age 62.) “I just wish it was longer.” (Male head and neck cancer survivor, age 58.) “I could certainly have gotten on the subway and made the trek downtown but I just like the convenience and for me it’s just more practical to be able to do them at home.” (Female breast cancer survivor, age 65.)
	Some participants appreciated having a non-group option, as they didn’t want to be a part of a group session due to the lack of privacy. Finally, some participants had no issue attending the in-person assessment appointments as they held flexible jobs and had flexible schedules. Others found that it was challenging to come to the hospital for midday assessment appointments and suggested that a wider range of time slots be offered to participants	“I feel like I’m eavesdropping when we’re in a group and they’re telling their heart and soul yeah and I always feel like it was none of my business to be listening. […] I always felt sort of awkward.” (Male head and neck cancer survivor, age 58.) “I think some people might find coming in to do the physical assessment might be tricky if they are working and they can’t take the time so fortunately my office is very supportive and that wasn’t an issue for me but um yea it might help if those, the, in person appointments could happen you know either earlier in the morning before people start or sort of much later in the day, at the end of the day.” (Female gynecological cancer survivor, age 53.)

**Table 4:** Qualitative Table for Acceptability.

Category	Analytic Note	Example Quote
Satisfaction with the CaRE@Home Program	All interviewed participants were very satisfied with the program and liked how it was organized and structured. They appreciated the support that they received during their program participation. They liked the various topics that were covered, notably the nutrition resources.	“I thought it was very well organized in the sense that it helps put together a number of things which seem basic and things we should know about taking care of ourselves.” (Female breast cancer survivor, age 51.)
	Participants appreciated that the modules could provide interesting and important information, yet struggled to find time to complete them and to implement the new skills into their daily lives. They would often complete the first few topics, but their engagement would decline as the weeks progressed and their lives became busy.	“I thought the modules were great they were very helpful but as I said for example with when you just read stuff yes it’s very difficult to actually implement.” (Female breast cancer survivor, age 62.)
	Health coaching calls were a valuable program component that provided the much-needed human touch element. Participants also reported that the health coaching calls provided structure and support during a time when they often felt alone. The calls also made a few participants realize that they did in fact need the additional support, even though they were not necessarily at first aware.	“I always enjoyed the check-in because it was an opportunity to share insights or ask questions and clarify things. […] I think it’s really extremely extremely valuable and I greatly enjoyed [them].” (Female breast cancer survivor, age 65.) “And the weekly call-in with the kinesiologist. That was also a great motivator, you know, because if I was just given the workbook and the e-classes I’m not sure if I would have stuck with the program, you know, dutifully. Touching base with someone, that was really helpful.” (Female gynecological cancer survivor, age 53.)
	Participants liked how the Fitbit^TM^ and the Physitrack^®^ technologies motivated them and allowed them to easily see their exercise prescription and progression while at home. They also appreciated the added element of having video tutorials, as well as having their kinesiologists be able to make remote weekly updates to their plans.	“It (Fitbit™) really motivates me and I told him a story about how when at night I noticed I was still short about a hundred steps to hit that magic 10000 it was 11:30 at night and I just walked around the house till I got it so it’s a good thing. I really I love my Fitbit™.” (Female breast cancer survivor, age 65.) “First it (Physitrack^®^) tells you how to do the exercises if I’ve forgotten how. So yes it’s like a personal trainer. It’s a great little thing and who doesn’t love checking a box right? And saying you’re done for the day. It’s an endorphin fix right there. It’s nice seeing a great big green tick - like well done.” (Male lymphoma cancer survivor, age 37.)
Benefits of the Program	Participants spoke about the benefits of participating in the CaRE@Home program, including managing a cancer diagnosis and taking care of yourself.	“I am eternally grateful that I was part of this program. I don’t know where I would have been in my recovery from cancer had I not joined the program.” (Female breast cancer survivor, age 57.)
	Many talked about gaining a new appreciation for exercise. They realized that exercise could minimize their symptoms and improve their sleep and/or fatigue. Some participants also learned how to realistically fit exercise into their daily and weekly schedules. Some participants liked that their exercise plan could be modified as the weeks progressed, allowing for additional challenges as they became stronger and/or their functional abilities changed.	“Yea, I just wanted to be stronger you know. I definitely accomplished that. Being assigned fitness exercises and the regular check-in, that helped me keep on track.” (Female gynecological cancer survivor, age 53.) “When that 2 o’clock slump came I worked out and was energized for the rest of the day. Yea. And I used the exercises. To be less fatigued.” (Male lymphoma cancer survivor, age 37.) “I get pretty regular sleep now. I think part of it was the exercise helps. But also part of it was letting go of some things.” (Female breast cancer survivor, age 51.)
Areas for program improvements	Some participants felt that the e-modules were too long and others preferred to be able to complete them on their own timeline rather than a prescribed weekly frequency. Others did not seem to be interested in the e-modules and only wanted to focus on the exercise component of the intervention.	“So the online education thing didn’t really work out well for me at all. But I know yeah it’s unfortunate. It basically just came down to me not having time to do it.” (Male lymphoma cancer survivor, age 37.) “I do think there were times where it would have helped me just have a bit more time to self-reflect on that topic as opposed to moving on to another topic.” (Female breast cancer survivor, age 51.)
	Participants spoke about the timing of the program and some suggested that it would have been more helpful if they had received it earlier, during treatment, as opposed to after treatment ended.	“If those modules could be made available to people as they’re going through their treatment.” (Female breast cancer survivor, age 51.)
	Participants suggested having a train-the-trainer model with remote kinesiologists who could do the assessments in the community rather than having patients come to downtown Toronto.	“If you can look into having remote locations for people to go to instead of coming to downtown Toronto great. It is possible that we will take advantage of. “(Female breast cancer survivor, age 57.)
	Participants suggested that some of the health coaching phone check-ins could be done over video conferencing to allow for a more personal touch and to demonstrate exercises etc.	“Rather than phone calls what about doing something where you’re skyping or you can actually see so it’s still remote, but yeah, they would be able to see you and you would see them.” (Female breast cancer survivor, age 57.)
	Participants suggested adding in some content and support for the patient’s caregiver.	“I think that it would have been cool that there was one module that included your main support person because they’re going through it too but from a different angle.”(Female breast cancer survivor, age 51.)

**Table 5:** Patient-Reported outcomes (PRO’s).

Outcome Measure	Time Point	*n*	Mean (SE)	95% CI	Difference in the Estimates (SE)	*p*-Value
WHODAS	T1	33	9.84(1.14)	7.52–12.2	-	-
	T2	29	8.17(1.01)	6.13–10.2	−1.66(0.70)	0.03
	T3	26	7.56(1.10)	5.32–9.80	−2.28(0.81)	0.008
ESAS Measures						
Pain	T1	35	1.94(0.36)	1.21–2.67	-	-
	T2	29	2.18(0.39)	1.37–2.98	−0.23(0.44)	0.598
	T3	28	1.81(0.38)	1.03–2.59	−0.13(0.35)	0.719
Tiredness	T1	35	3.71(0.41)	2.88–4.55	-	-
	T2	29	3.15(0.41)	2.31–3.99	−0.56(0.34)	0.105
	T3	28	3.16(0.35)	2.45–3.87	−0.55(0.29)	0.072
Drowsiness	T1	35	2.17(0.40)	1.36–2.98	-	-
	T2	29	2.15(0.35)	1.43–2.86	−0.02(0.37)	0.948
	T3	28	2.16(0.41)	1.33–2.99	−0.01(0.47)	0.984
Depression	T1	35	2.08(0.39)	1.28–2.89	-	-
	T2	29	1.90(0.39)	1.09–2.70	−0.18(0.26)	0.478
	T3	28	1.82(0.37)	1.05–2.57	−0.27(0.27)	0.323
Anxiety	T1	35	2.54(0.47)	1.58–3.50	-	-
	T2	29	2.23(0.42)	1.38–3.08	−0.31(0.33)	0.202
	T3	28	2.06(0.44)	1.18–2.95	−0.48(0.37)	0.355
Wellbeing	T1	35	3.14(0.39)	2.33–3.95	-	-
	T2	29	3.41(0.46)	2.47–4.34	0.27(0.43)	0.808
	T3	28	3.42(0.41)	2.58–4.26	0.28(0.26)	0.527
GODIN						
Total LSI	T1	35	18.4(2.22)	13.9–22.9	-	-
	T2	31	28.4(3.37)	21.5–35.2	9.96(3.45)	0.007
	T3	26	30.5(3.30)	23.8–37.2	12.14(2.95)	0.0002
Moderate to strenuous LSI	T1	35	9.26(1.74)	5.73–12.8	-	-
	T2	27	18.6(3.02)	12.5–24.7	9.34(2.91)	0.003
	T3	25	20.0(3.33)	13.2–26.8	10.8(3.17)	0.002
Work Status						
During the past seven days, how many hours did you miss from work because of problems associated with cancer diagnosis and treatment?	T1	16	4.83(1.52)	1.66–8.01	-	-
	T2	16	2.31(0.94)	0.36–4.27	−2.52(1.06)	0.069
	T3	17	1.88(0.79)	0.22–3.53	−2.96(1.27)	0.074
During the past seven days, how much did your cancer diagnosis and treatment affect your productivity while you were working?	T1	15	3.44(0.53)	2.33–4.54	-	-
	T2	16	2.62(0.65)	1.27–3.96	−0.82(0.66)	0.438
	T3	14	2.03(0.48)	1.03–3.03	−1.41(0.49)	0.026

**Table 6:** Physiological Outcomes.

Outcome Measure	Time Point	*n*	Mean (SE)	95% CI	Difference in theEstimates (SE)	*p*-Value
Six Minute Walk Test(Meters)	T1	35	469.0(16.72)	435.0–502.9	-	-
	T2	30	510.9(13.74)	483.0–538.9	41.96(8.09)	<0.001
	T3	25	502.5(19.02)	463.8–541.1	33.48(12.4)	0.010
Grip Strength(Kg)	T1	35	58.5(3.57)	51.22–65.75	-	-
	T2	31	61.6(3.59)	54.31–68.92	3.13(0.99)	0.003
	T3	27	64.1(3.59)	56.79–71.39	5.61(1.16)	<0.001
Heart Rate(Beats per minute)	T1	35	70.9(1.74)	67.34–74.44	-	-
	T2	31	71.7(1.36)	68.90–74.43	0.78(1.47)	0.597
	T3	27	73.0(1.39)	67.36–74.44	2.06(1.56)	0.197
Systolic Blood Pressure (mm Hg)	T1	35	122.3(2.09)	118.0–126.6	-	-
	T2	31	120.3(2.78)	114.6–125.9	−2.02(2.25)	0.377
	T3	27	118.3(4.26)	109.6–126.9	−3.98(4.58)	0.391
Diastolic Blood Pressure (mm Hg)	T1	35	80.7(2.98)	74.65–86.78	-	-
	T2	31	76.7(1.63)	73.35–79.99	−4.05(2.99)	0.186
	T3	27	77.2(1.32)	74.52–79.90	−3.50(3.18)	0.279
BMI(kg/m^2^)	T1	34	27.5(1.02)	25.38–29.53	-	-
	T2	29	27.5(1.04)	25.41–29.67	0.08(0.10)	0.417
	T3	26	27.7(1.07)	25.38–29.53	0.19(0.17)	0.267
