# Addendum: MacDonald, A.M., et al. CaRE @ Home: Pilot Study of an Online Multidimensional Cancer Rehabilitation and Exercise Program for Cancer Survivors. *J. Clin. Med.* 2020, *9*, 3092

**DOI:** 10.3390/jcm9113440

**Published:** 2020-10-26

**Authors:** Anne Marie MacDonald, Aleksandra Chafranskaia, Christian J. Lopez, Manjula Maganti, Lori J. Bernstein, Eugene Chang, David Michael Langelier, Maya Obadia, Beth Edwards, Paul Oh, Jacqueline L. Bender, Shabbir M.H. Alibhai, Jennifer M. Jones

**Affiliations:** 1 Cancer Rehabilitation and Survivorship Program, Princess Margaret Cancer Centre, Toronto, ON M5G 2C1, Canada; annemarie.hospod@uhnresearch.ca (A.M.M.); aleksandra.chafranskaia@uhn.ca (A.C.); christian.lopez@uhnresearch.ca (C.J.L.); lori.bernstein@uhn.ca (L.J.B.); eugene.chang@uhn.ca (E.C.); david.langelier@uhn.ca (D.M.L.); maya.obadia@uhnresearch.ca (M.O.); beth.edwards@uhnresearch.ca (B.E.); jackie.bender@uhnresearch.ca (J.L.B.); 2 IMS Program, Faculty of Medicine, University of Toronto, Toronto, ON M5S 1A8, Canada; 3 Department of Physical Therapy, University of Toronto, Toronto, ON M5G 1V7, Canada; 4 Department of Biostatistics, Princess Margaret Cancer Centre, Toronto, ON M5G 2C1, Canada; Manjula.Maganti@uhn.ca; 5 Department of Psychiatry, Faculty of Medicine, University of Toronto, Toronto, ON M5S 1A8, Canada; 6 Department of Medicine, University of Toronto, Toronto, ON M5S 1A8, Canada; paul.oh@uhn.ca (P.O.); shabbir.alibhai@uhn.ca (S.M.H.A.); 7 Department of Supportive Care, Princess Margaret Cancer Centre, University Health Network, Toronto, ON M5G 2C1, Canada

The authors wish to make the following corrections to this paper [1].

The authors would like to add Dr. Daniel Santa Mina as a co-author to the paper given his important contributions to the development of the CaRE @ Home intervention and the study methods.

The original list of authors is:

Anne Marie MacDonald ^1,2^, Aleksandra Chafranskaia ^1,3^, Christian J. Lopez ^1^, Manjula Maganti ^4^, Lori J. Bernstein ^1,5^, Eugene Chang ^1,6^, David Michael Langelier ^1,6^, Maya Obadia ^1^, Beth Edwards ^1^, Paul Oh ^6^, Jacqueline L. Bender ^1^, Shabbir M.H. Alibhai ^6,7^ and Jennifer M. Jones ^1,5,^*

^1^  Cancer Rehabilitation and Survivorship Program, Princess Margaret Cancer Centre, Toronto, ON M5G 2C1, Canada; annemarie.hospod@uhnresearch.ca (A.M.M.); aleksandra.chafranskaia@uhn.ca (A.C.); christian.lopez@uhnresearch.ca (C.J.L.); lori.bernstein@uhn.ca (L.J.B.); eugene.chang@uhn.ca (E.C.); david.langelier@uhn.ca (D.M.L.); maya.obadia@uhnresearch.ca (M.O.); beth.edwards@uhnresearch.ca (B.E.); jackie.bender@uhnresearch.ca (J.L.B.)
^2^  IMS Program, Faculty of Medicine, University of Toronto, Toronto, ON M5S 1A8, Canada
^3^  Department of Physical Therapy, University of Toronto, Toronto, ON M5G 1V7, Canada
^4^  Department of Biostatistics, Princess Margaret Cancer Centre, Toronto, ON M5G 2C1, Canada; Manjula.Maganti@uhn.ca
^5^  Department of Psychiatry, Faculty of Medicine, University of Toronto, Toronto, ON M5S 1A8, Canada
^6^  Department of Medicine, University of Toronto, Toronto, ON M5S 1A8, Canada; paul.oh@uhn.ca (P.O.); shabbir.alibhai@uhn.ca (S.M.H.A.)
^7^  Department of Supportive Care, Princess Margaret Cancer Centre, University Health Network, Toronto, ON M5G 2C1, Canada

And should be replaced with:

Anne Marie MacDonald ^1,2^, Aleksandra Chafranskaia ^1,3^, Christian J. Lopez ^1^, Manjula Maganti ^4^, Lori J. Bernstein ^1,5^, Eugene Chang ^1,6^, David Michael Langelier ^1,6^, Maya Obadia ^1^, Beth Edwards ^1^, Paul Oh ^6^, Jacqueline L. Bender ^1^, Shabbir M.H. Alibhai ^6,7^, Daniel Santa Mina ^8^ and Jennifer M. Jones ^1,5,^*

^1^  Cancer Rehabilitation and Survivorship Program, Princess Margaret Cancer Centre, Toronto, ON M5G 2C1, Canada; annemarie.hospod@uhnresearch.ca (A.M.M.); aleksandra.chafranskaia@uhn.ca (A.C.); christian.lopez@uhnresearch.ca (C.J.L.); lori.bernstein@uhn.ca (L.J.B.); eugene.chang@uhn.ca (E.C.); david.langelier@uhn.ca (D.M.L.); maya.obadia@uhnresearch.ca (M.O.); beth.edwards@uhnresearch.ca (B.E.); jackie.bender@uhnresearch.ca (J.L.B.)
^2^  IMS Program, Faculty of Medicine, University of Toronto, Toronto, ON M5S 1A8, Canada
^3^  Department of Physical Therapy, University of Toronto, Toronto, ON M5G 1V7, Canada
^4^  Department of Biostatistics, Princess Margaret Cancer Centre, Toronto, ON M5G 2C1, Canada; Manjula.Maganti@uhn.ca
^5^  Department of Psychiatry, Faculty of Medicine, University of Toronto, Toronto, ON M5S 1A8, Canada
^6^  Department of Medicine, University of Toronto, Toronto, ON M5S 1A8, Canada; paul.oh@uhn.ca (P.O.); shabbir.alibhai@uhn.ca (S.M.H.A.)
^7^  Department of Supportive Care, Princess Margaret Cancer Centre, University Health Network, Toronto, ON M5G 2C1, Canada
^8^  Faculty of Kinesiology and Physical Education, University of Toronto, Toronto, ON M5S 2W6, Canada; daniel.santamina@utoronto.ca

The authors would like to apologize for this omission from the original manuscript. These changes do not affect the scientific results.

## References

[1] MacDonald A.M., Chafranskaia A., Lopez C.J., Maganti M., Bernstein L.J., Chang E., Langelier D.M., Obadia M., Edwards B., Oh P. (2020). CaRE @ Home: Pilot Study of an Online Multidimensional Cancer Rehabilitation and Exercise Program for Cancer Survivors. J. Clin. Med..

